# Supplementary material for: Invariance to background noise as a signature of non-primary auditory cortex
Source: Nat Commun. 2019 Sep 2;10:3958. doi: 10.1038/s41467-019-11710-y (PMC6718388; doi:10.1038/s41467-019-11710-y)
Supplement: Supplementary file 1 — Supplementary Information [file 41467_2019_11710_MOESM1_ESM.pdf]

Supplementary Information

Invariance to background noise as a signature of non-primary auditory cortex

Alexander J. E. Kell[1,2,3,5], Josh H. McDermott[1,2,3,4]

1. Department of Brain and Cognitive Sciences, MIT
2. McGovern Institute for Brain Research, MIT
3. Center for Brains, Minds, and Machines, MIT
4. Program in Speech and Hearing Biosciences and Technology, Harvard University
5. Present address: Zuckerman Institute of Mind, Brain, and Behavior, Columbia University

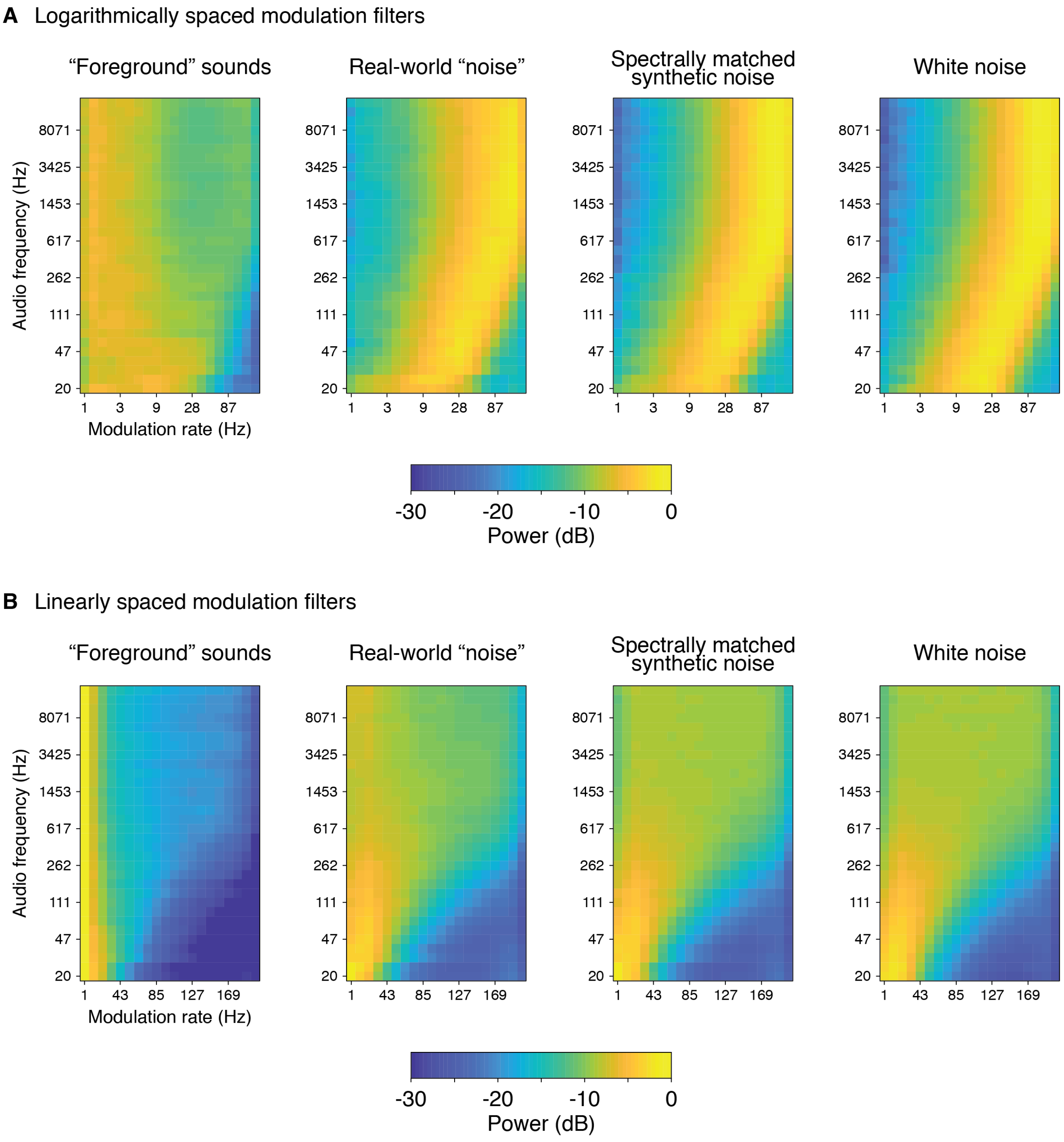

Supplementary Figure 1. Modulation power in stimuli.

(A) Each panel shows the mean modulation power in each of a bank of bandpass modulation filters for a set of stimuli (the right-most column, white noise, was not used as a stimulus in the study but is included as a structureless reference). Audio waveforms were passed through a bank of bandpass cochlear filters, and the Hilbert envelope of each filter was passed through a second bank of (modulation) filters. These modulation filters were logarithmically spaced and tiled the modulation domain from 1-200 Hertz (Hz). Each bin indicates the average power for a modulation rate within a cochlear channel. Power is expressed in decibels (dB) and the color scale, which is identical across all four panels, is normalized such that the max across all panels is set to zero.

(B) Same as (A) but with linearly spaced modulation filters. This alternative filter bank is less consistent with what is known about biological auditory systems, but makes the differences between white noise and the real-world noise we used more visible. Filters tile the same range (1-200 Hz) as (A).

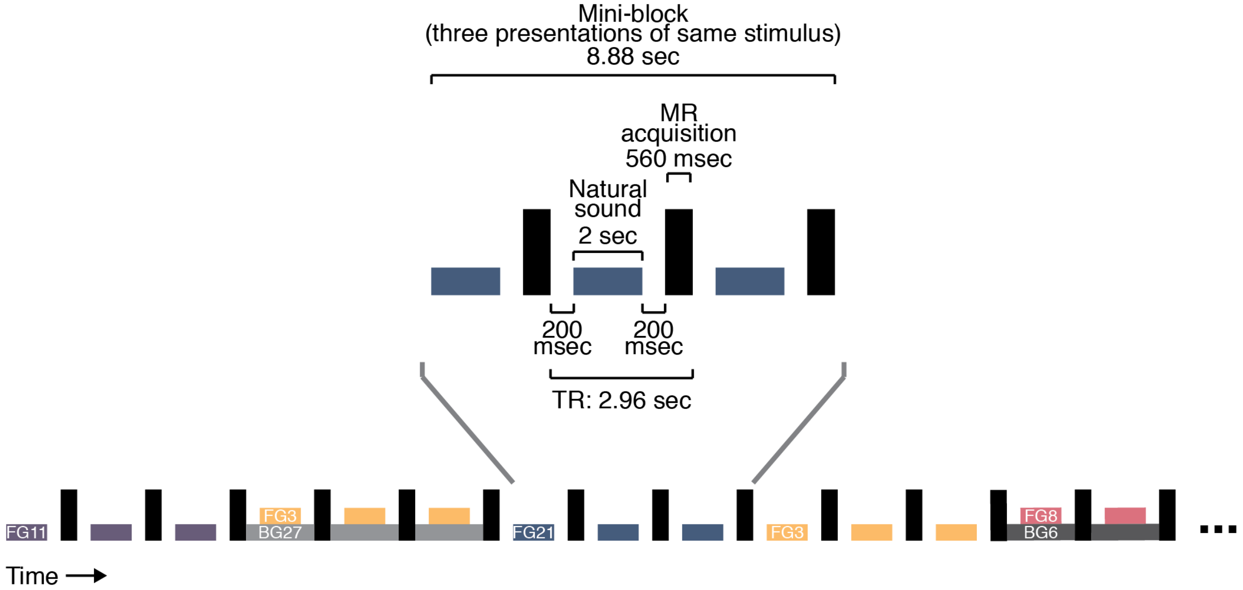

Supplementary Figure 2. Schematic of stimulus presentation and experimental design.
We used a sparse scanning paradigm, with MR acquisitions interleaved with stimulus presentation such that the noise produced by acquisitions did not overlap with stimulus presentation. The gap between acquisitions was 2.4 seconds (sec). In Experiments 1, 2, and 3, acquisitions lasted 560 milliseconds (msec). In Experiment 4, the acquisition lasted 870 milliseconds—to measure responses in visual cortex during, we increased the number of slices such that coverage extended over the entirety of the occipital lobe. The TR was therefore 2.96 seconds in Experiments 1, 2, and 3, and 3.27 seconds in Experiment 4. Stimuli were presented in a mini-block design, wherein the same stimulus was presented three times in a row. In pilot experiments, this design was found to yield more reliable BOLD responses given a fixed amount of scan time than an event-related design (mini-blocks of 1 stimulus presentation) or a design with five presentations per mini-block. "Foreground" sounds were 2 seconds and background noises (both real-world and synthetic) were 2.4 seconds; they are schematically represented by "FG" or "BG" in the figure. For mixtures of sounds and noise, the onset of the foreground sound was offset from the onset of the noise, so as to diminish the chance of perceptual grouping.

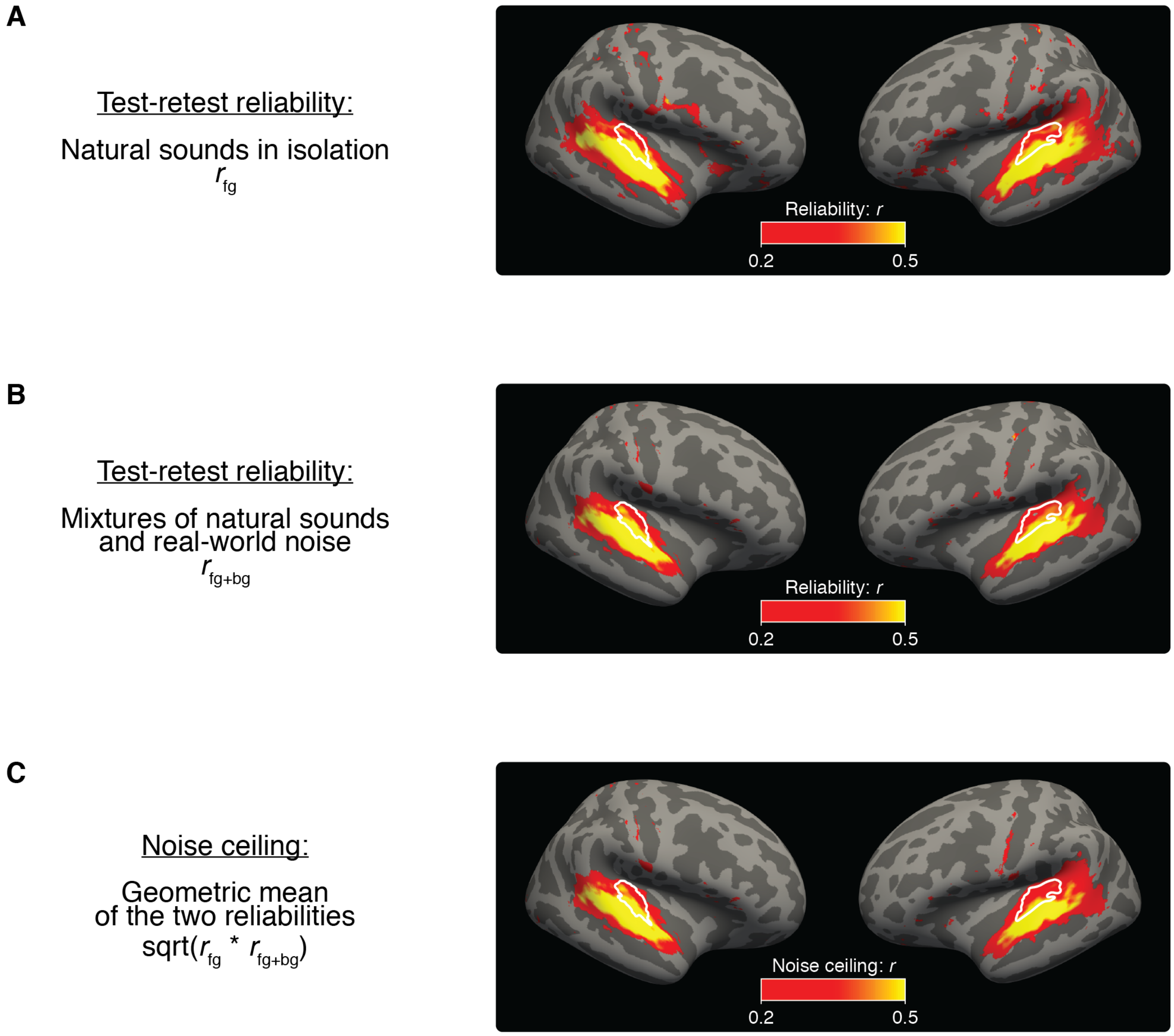

Supplementary Figure 3. Reliability of cortical responses.

(A) Map of the test-retest reliability of voxel responses to natural sounds in isolation from Experiment 1. Reliability was computed in individual subjects, and then averaged across all eleven subjects. Only voxels with an average correlation coefficient greater than 0.2 are assigned a color. For reference, the white outline indicates the primary auditory cortical region of interest (TE 1.1 and 1.0); “fg” stands for “foreground”.

(B) Map of the test-retest reliability of responses to foreground-background mixtures. Plotting conventions, including color scale, are the same as (A); “bg” stands for “background”.

(C) Map of the maximum possible correlation (i.e., the noise ceiling) between responses to foregrounds sounds and foreground-background mixtures, which is simply the geometric mean of the values shown in (A) and (B). Plotting conventions, including color scale, are the same as (A) and (B).

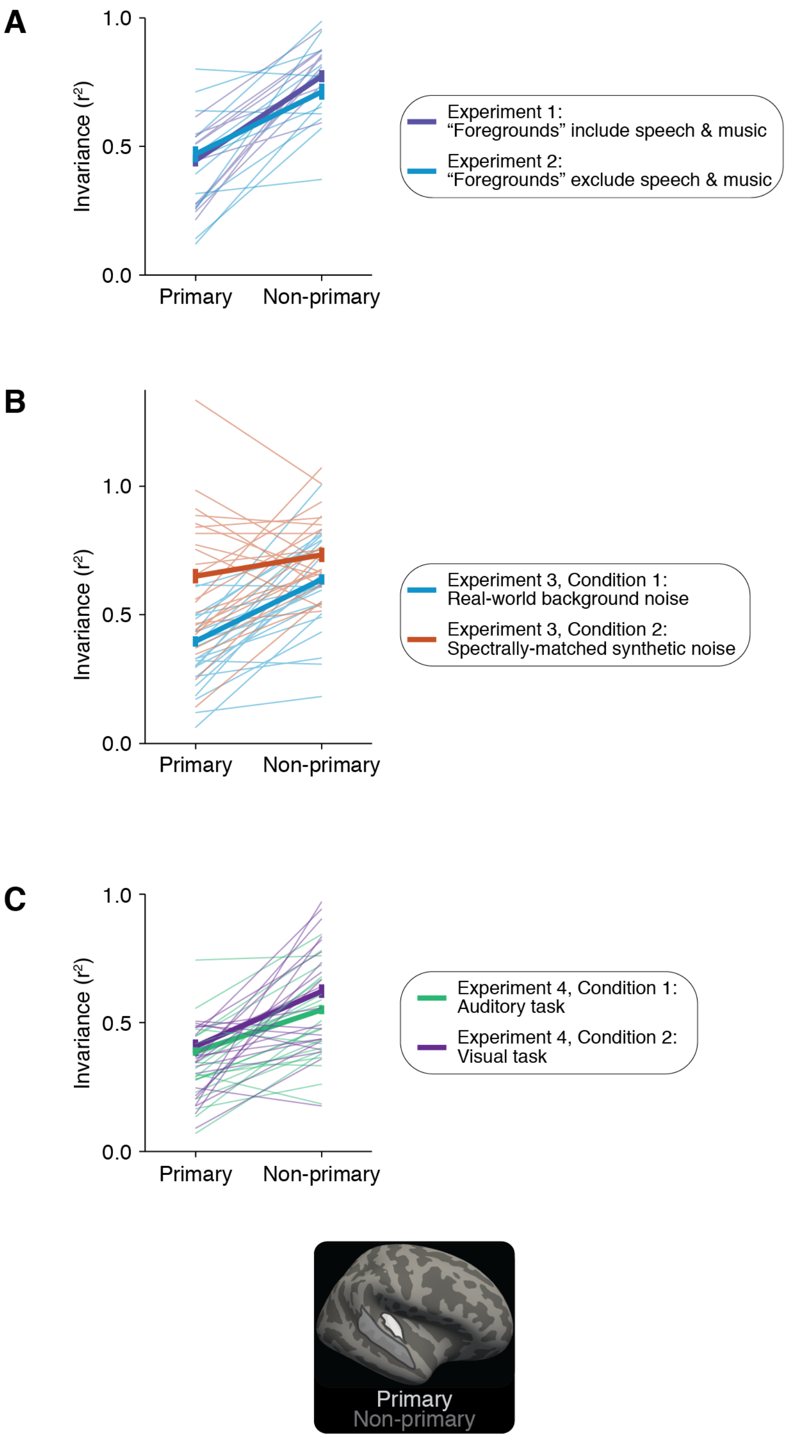

Supplementary Figure 4. Invariance measured in individual participants.

(A) Each line indicates invariance in an individual participant from Experiment 1 or 2. Bold lines indicate mean and within-subject SEM.

(B) Each line indicates invariance in an individual participant in one condition from Experiment 3. Bold lines indicate mean and within-subject SEM.

(C) Each line indicates invariance in an individual participant in one condition from Experiment 4. Bold lines indicate mean and within-subject SEM.

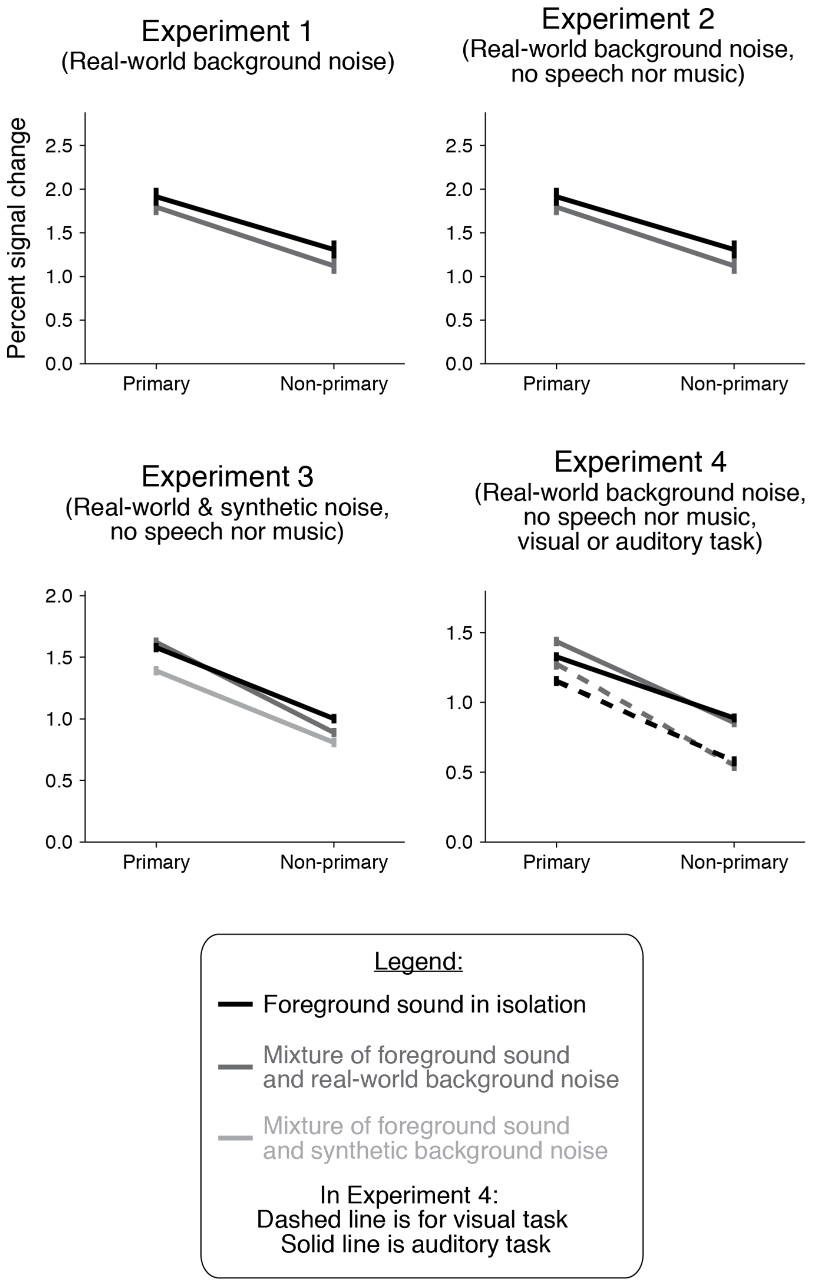

Supplementary Figure 5. Mean responses to foregrounds and mixtures in primary and non-primary regions.
Mean response in percent signal change in the primary and non-primary regions of interest. Error bars reflect within-subject SEMs.

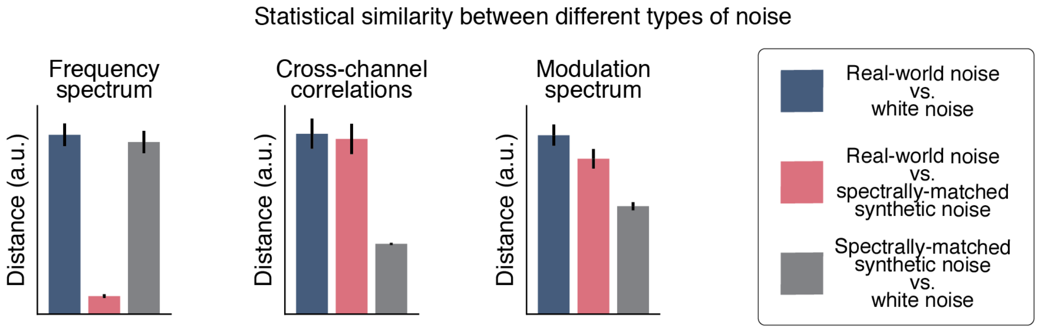

Supplementary Figure 6. Statistical similarity between types of noise.
Average Euclidean distance in statistics between real-world noise and the corresponding spectrally-matched synthetic noise. For reference, we have included distances between each of these noises and white noise, which was not included as a stimulus, but serves here as a structureless reference. As expected, both real-world and spectrally-matched synthetic noise have similar frequency spectra (left), both of which are dissimilar from white noise. By contrast, the spectrally matched synthetic noise has cross-channel correlations (middle) and modulation spectra (right) that are much more similar to white noise than to real-world noises. Error bars plot SEMs across sounds; “a.u.” denotes “arbitrary units”.

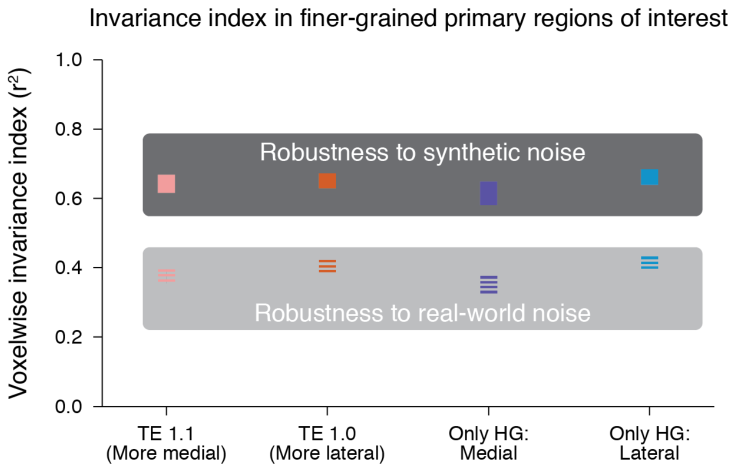

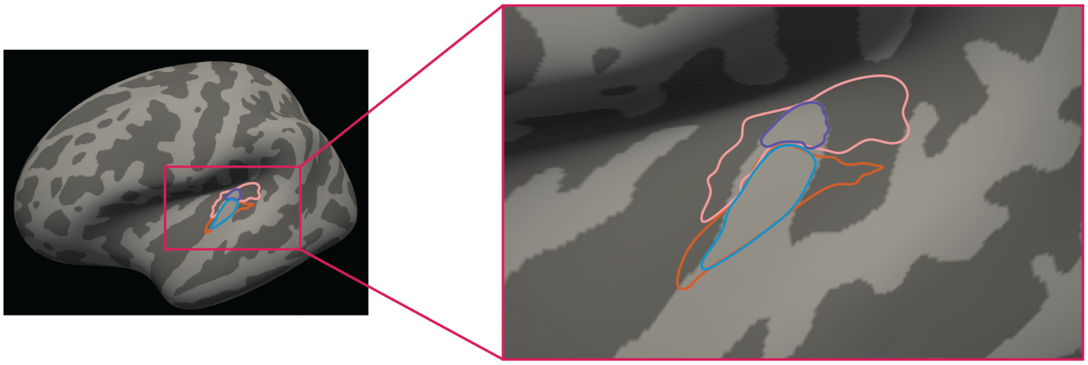

Supplementary Figure 7. Robustness in more fine-grained primary regions of interest.
Top: Mean invariance index in regions of interest in and around Heschl's gyrus (HG) from Experiment 3. From left to right: TE 1.1, TE 1.0, the portion of TE 1.1 within Heschl's gyrus, and the portion of TE 1.0 within Heschl's gyrus. Vertical dimension of symbols indicates within-subject SEMs. The invariance index was not significantly different between TE 1.1 and 1.0 (two-tailed paired t test for real-world noise: $t_{18} = 0.66$, $p = 0.518$; for synthetic noise: $t_{18} = 0.47$, $p = 0.689$) or the sub-regions constrained to lie within Heschl's gyrus (two-tailed paired t test for real-world noise: $t_{18} = 1.39$, $p = 0.182$; for synthetic noise: $t_{18} = 1.22$, $p = 0.240$). Bottom left: Lateral view of inflated brain with ROIs. Bottom right: Zoomed-in view of ROIs.

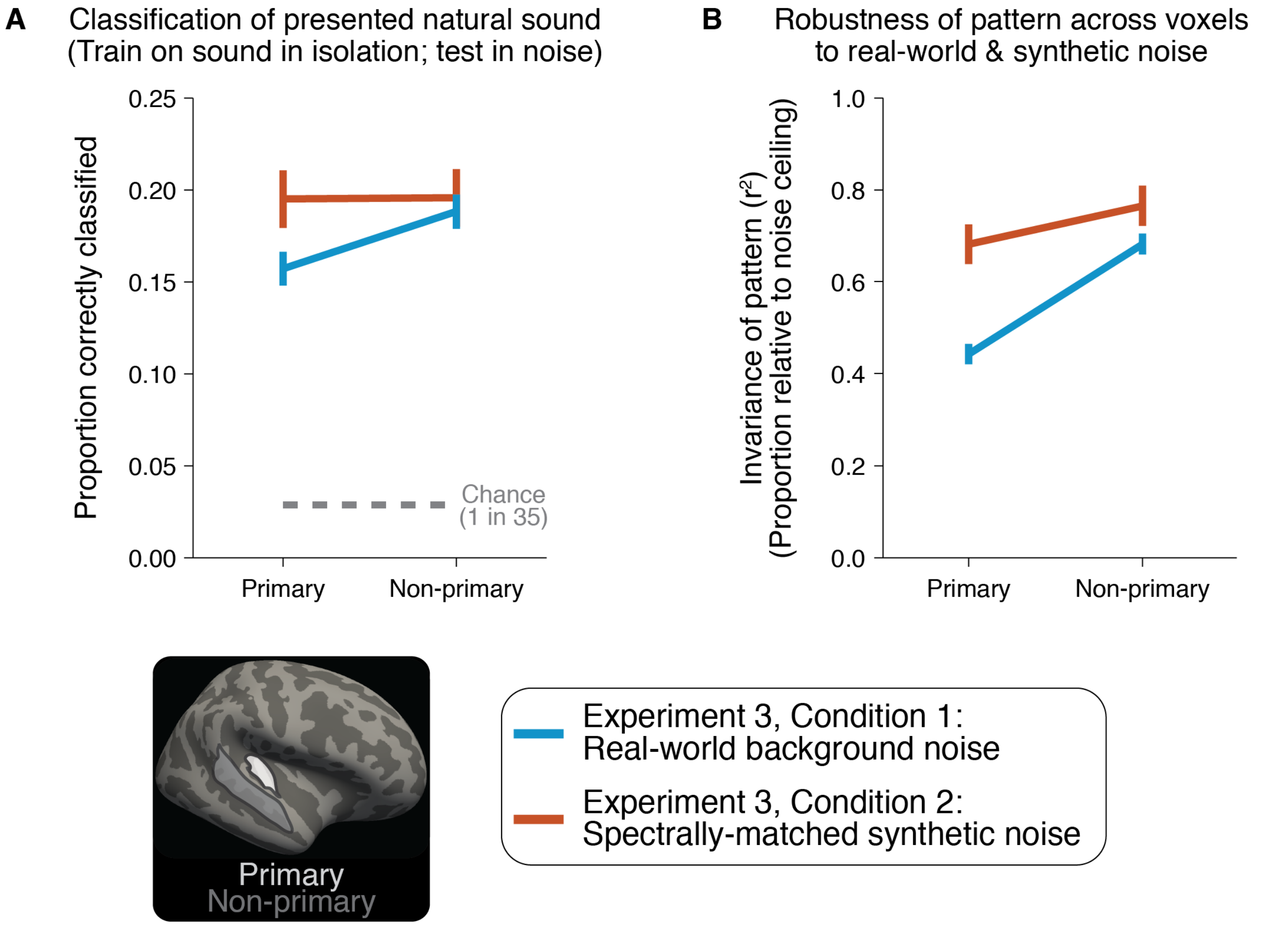

Supplementary Figure 8. The robustness of the patterns across voxels is similar to that of individual voxels.

(A) Proportion of stimuli correctly classified from multivoxel patterns of natural sounds from Experiment 3. Nearest-neighbor classifiers, with Pearson correlation as the distance metric, were trained on foreground sounds in isolation and tested on foreground sounds in noise (either real-world or synthetic), separately for primary and non-primary regions of interest.

(B) Dashed gray line indicates chance performance in the 35-way classification. Classification was significantly better in non-primary areas than primary areas for real-world noise (two-tailed paired t test: t_{22} = 2.42, p = 0.0244), but not synthetic noise (two-tailed paired t test: t_{22} = 0.0286, p = 0.977). Error bars plot within-subject SEMs.

(C) Invariance of the pattern of responses in primary and non-primary regions to real-world and synthetic noise. The values are normalized by the maximum possible value given the reliability of the patterns. Error bars plot within-subject SEMs.

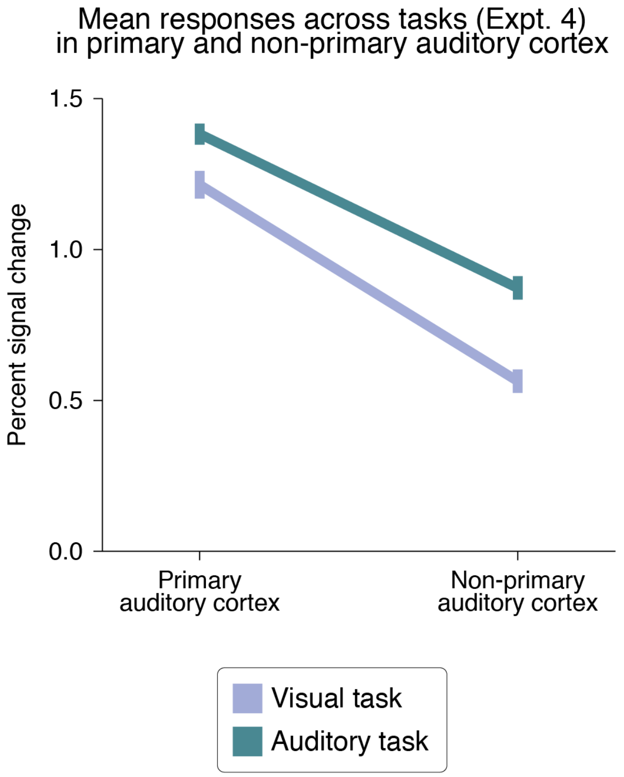

Supplementary Figure 9. Mean responses in primary and non-primary auditory ROIs across tasks. Mean responses in primary and non-primary auditory cortex during the visual and auditory tasks (Experiment 4). Error bars reflect within-subject SEMs.

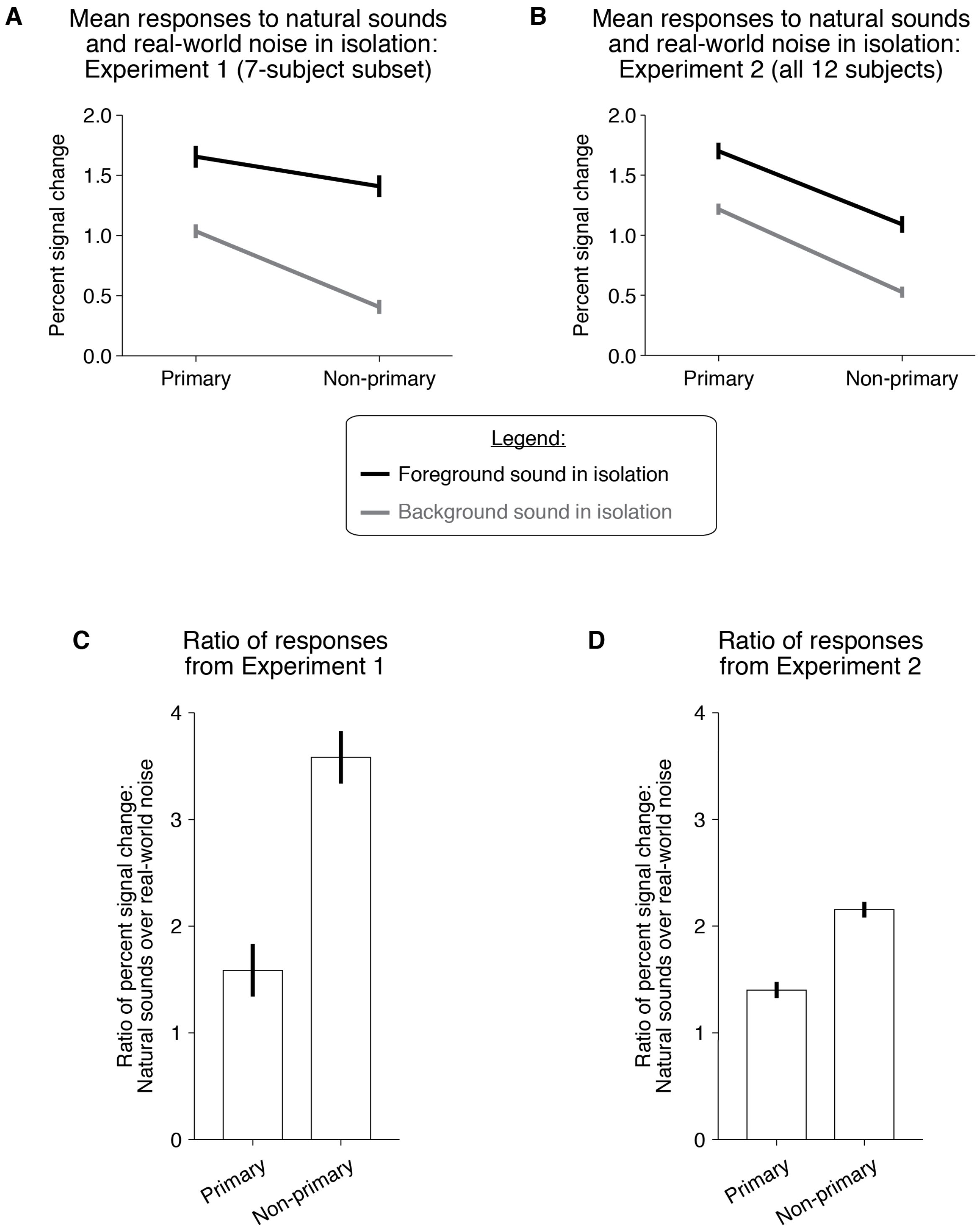

Supplementary Figure 10. Mean responses to foreground and background sounds in isolation.

(A) Mean responses to foreground sounds and real-world background noise in primary and non-primary regions from Experiment 1. Only seven of eleven subjects in Experiment 1 were presented with the background noise sounds in isolation (i.e., not superimposed on the foreground sounds). Error bars plot within-subject SEMs.

(B) Same as (A) but for Experiment 2. Error bars plot within-subject SEMs.

(C) Ratio of responses the means from (A). Error bars reflect within-subject SEMs.

(D) Ratio of means from (B). Error bars plot within-subject SEMs.

“Foreground” sounds

- Knives sharpening
- Peeling a potato
- Cracking knuckles
- Car accelerating
- Lion growling
- Cat meowing
- Nose blowing
- Chicken clucking
- Reception desk bell ringing
- Coughing
- Opening a can
- Mooing a cow
- Pinball machine
- Person sobbing
- Race car driving
- Dialup modem
- Rooster crowing
- Rolling dice
- Sheep bah-ing
- Dishes clanking
- Person sighing
- Puppy whining
- Sipping a beverage
- Baby crying
- Rock splashing in water
- Doorbell ringing
- Spoon scraping bowl
- Toilet flushing
- Stapler stapling
- Hawk screech
- Bowling pins colliding
- Horse neighing
- Vacuum
- Ice machine
- Writing on paper

Real-world “background noise”

(Note: in Experiment 3, Condition 2 these were replaced with spectrally-matched Gaussian noise.)

- AM radio static
- Air conditioner
- Lawn mower
- Swamp insects
- Forest fire
- Frying bacon
- Idling boat
- Bubbling water
- Disposal
- Frying eggs
- Construction site
- Blender
- Radio static
- Waterfall
- Industrial presses
- Polite appluase
- Crowd noise
- Jackhammer
- Fire in a room
- Frogs
- Pneumatic drills
- Pouring coffee beans
- Printing press
- Edger
- Crowd in auditorium
- Wind moaning
- Wind whistling
- Small river
- Applause in auditorium
- Ambient large railway station
- Heavy rain
- Sander rattling
- Crowd in theater
- Train chugging
- Metal lathe

Supplementary Table 1. List of all sounds in Experiment 2, Experiment 3, and Experiment 4.
Top: List of foreground sounds, none of which were examples of speech nor music. The same foreground sounds were presented in Experiment 2, Experiment 3, and Experiment 4. Bottom: List of background noises. In Experiment 2 and Experiment 4, the real-world clips were presented; in Experiment 3, each clip was replaced with a Gaussian noise signal whose spectrum was shaped to match the long-term average spectrum of each background noise.
