# Supplementary material for: Invariance to background noise as a signature of non-primary auditory cortex
Source: Nat Commun. 2019 Sep 2;10:3958. doi: 10.1038/s41467-019-11710-y (PMC6718388; doi:10.1038/s41467-019-11710-y)
Supplement: Supplementary file 3 — Reporting Summary [file 41467_2019_11710_MOESM3_ESM.pdf]

nature research

Corresponding author(s): Alexander Kell (alexkell@mit.edu, alex.kell@columbia.edu)

Last updated by author(s): Jul 17, 2019

Reporting Summary

Nature Research wishes to improve the reproducibility of the work that we publish. This form provides structure for consistency and transparency in reporting. For further information on Nature Research policies, see Authors & Referees and the Editorial Policy Checklist.

Statistics

For all statistical analyses, confirm that the following items are present in the figure legend, table legend, main text, or Methods section.

n/a	Confirmed	
☐	☒	The exact sample size (*n*) for each experimental group/condition, given as a discrete number and unit of measurement
☐	☒	A statement on whether measurements were taken from distinct samples or whether the same sample was measured repeatedly
☐	☒	The statistical test(s) used AND whether they are one- or two-sided<br>*Only common tests should be described solely by name; describe more complex techniques in the Methods section.*
☐	☒	A description of all covariates tested
☐	☒	A description of any assumptions or corrections, such as tests of normality and adjustment for multiple comparisons
☐	☒	A full description of the statistical parameters including central tendency (e.g. means) or other basic estimates (e.g. regression coefficient) AND variation (e.g. standard deviation) or associated estimates of uncertainty (e.g. confidence intervals)
☐	☒	For null hypothesis testing, the test statistic (e.g. *F*, *t*, *r*) with confidence intervals, effect sizes, degrees of freedom and *P* value noted<br>*Give P values as exact values whenever suitable.*
☒	☐	For Bayesian analysis, information on the choice of priors and Markov chain Monte Carlo settings
☒	☐	For hierarchical and complex designs, identification of the appropriate level for tests and full reporting of outcomes
☐	☒	Estimates of effect sizes (e.g. Cohen's *d*, Pearson's *r*), indicating how they were calculated

Our web collection on statistics for biologists contains articles on many of the points above.

Software and code

Policy information about availability of computer code

Data collection	The Psychophysics toolbox (PsychToolbox: Brainard, 1997) was used for stimulus presentation and behavioral response recording.
Data analysis	Data analysis used: Python (2.7.14), numpy (1.9.3), scipy (0.15.1), Matlab (2013a), Freesurfer (5.3.0), and FSL (4.1).

For manuscripts utilizing custom algorithms or software that are central to the research but not yet described in published literature, software must be made available to editors/reviewers. We strongly encourage code deposition in a community repository (e.g. GitHub). See the Nature Research guidelines for submitting code & software for further information.

Data

Policy information about availability of data

All manuscripts must include a data availability statement. This statement should provide the following information, where applicable:

- Accession codes, unique identifiers, or web links for publicly available datasets
- A list of figures that have associated raw data
- A description of any restrictions on data availability

Data and the analysis code are available from corresponding author A.K. upon reasonable request.

Field-specific reporting

Please select the one below that is the best fit for your research. If you are not sure, read the appropriate sections before making your selection.

☒ Life sciences ☐ Behavioural & social sciences ☐ Ecological, evolutionary & environmental sciences

For a reference copy of the document with all sections, see nature.com/documents/nr-reporting-summary-flat.pdf

Life sciences study design

All studies must disclose on these points even when the disclosure is negative.

Sample size	Pilot versions of Experiment 1 indicated that the effect size of the difference in the mean of the invariance metric across voxels in each ROI was large. Indeed, there was a 90% chance of rejecting the null hypothesis that each participant's mean invariance in each ROI was are equal with just two participants, as evaluated with a paired two-tailed t-test with a p-value criterion (alpha) of 0.05. However, we also wanted to examine the invariance metric in maps across all of auditory cortex, and we wanted those maps to be reliable. Using pilot data (from the first four participants in Experiment 1), we estimated the split-half reliability of the maps (i.e., the correlation of the two maps derived from splits of the participant set) as a function of the number of participants using the Spearman-Brown correction. The predicted reliability ranged between r = 0.36 (n=2) to r = 0.89 (n=30). We chose a sample size of twelve participants, which yielded a projected reliability of r > 0.75, which we considered reasonable.
Data exclusions	One additional participant took part in Experiment 1, but was excluded because of high audiometric thresholds for one ear (35 dB HL). Four additional participants took part in Experiment 3 but were excluded: three did not complete the scanning session; the fourth completed the scanning session but had poor behavioral performance (mean performance on two alternative-forced choice in-scanner task: 48%; for other participants, mean performance: 90%, range: 85-97%). Five additional participants took part in Experiment 4: one was excluded because of their audiogram was suprathreshold (>15 dB HL for each ear), two others were excluded because they did not complete the scan session, and two were excluded for poor behavioral performance (less than 80% correct on the auditory task; for other participants, mean performance: 92%, range: 85-99%).
Replication	We found that non-primary areas were significantly more robust to real-world background noise than primary areas in Experiment 1, Experiment 2, Condition 1 in Experiment 3, and Condition 1 in Experiment 4.
Randomization	N/A - There were no experimental groups.
Blinding	Neither data collection nor analysis were performed blind to the conditions of the experiment.

Reporting for specific materials, systems and methods

We require information from authors about some types of materials, experimental systems and methods used in many studies. Here, indicate whether each material, system or method listed is relevant to your study. If you are not sure if a list item applies to your research, read the appropriate section before selecting a response.

Materials & experimental systems

n/a	Involved in the study	
☒	☐	Antibodies
☒	☐	Eukaryotic cell lines
☒	☐	Palaeontology
☒	☐	Animals and other organisms
☒	☐	Human research participants
☒	☐	Clinical data

Methods

n/a	Involved in the study	
☒	☐	ChIP-seq
☒	☐	Flow cytometry
☐	☒	MRI-based neuroimaging

Magnetic resonance imaging

Experimental design

Design type	Sounds were presented using a "mini-block" design. Each mini-block consisted of three presentations of the identical sound clip. After each sound, a single fMRI volume was collected, such that sounds were not presented simultaneously with the scanner noise ("sparse scanning"). See Supplementary Figure 2 for a schematic.
Design specifications	Foreground sounds were two-seconds long and were presented with 200 milliseconds of silence before and after each sound. Background noises were 2.4 seconds long. Mixtures were 2.4 seconds long, with the foreground starting 200 milliseconds after the start of the background and ending 200 milliseconds before the end of the background. Foregrounds and backgrounds had asynchronous onsets because common onsets are a well-established cue to perceptually group sounds, which we sought to avoid.<br><br>Each block lasted 8.88 seconds (three repetitions of a 2.96 second TR), except in Experiment 4 where each block lasted 9.81 seconds (three repetitions of a 3.27 second TR). This three-presentation block design was selected based on pilot experiments that showed that given the same amount of overall scan time, a three-presentation block gave more reliable BOLD responses than an event-related design (a "mini-block" of one) or a design with additional repetitions (e.g., with five presentations per block). Blocks were grouped into three runs (each run was ~6.5 minutes), with either thirty (Experiment 1), thirty-five (Experiment 2 and 3), or twenty-seven (Experiment 4) stimulus blocks presented in each run. In Experiment 1, Experiment 2, and Experiment 3, across three runs all stimuli would be presented exactly once; in Experiment 4, across four runs all stimuli would be presented once. In Experiments 1-3, each subject had twelve runs total across a single two-hour scanning session; in Experiment 4, sixteen runs were in the two-hour session.

Therefore, in all experiments each stimulus was presented four times. The order of stimuli was randomized across each set of runs. To enable estimation of the baseline response, silence blocks were included (~20% of TRs), which were the same duration as the stimulus blocks and were randomly interleaved throughout each run.

Behavioral performance measures

For Experiments 1-3 and Condition 1 in Experiment 4 (the auditory task), in each block one of the three presentations was 7 dB lower in level than the other two (the lower-intensity presentation was never the first sound). Subjects were instructed to press a button when they heard the lower intensity stimulus. Sounds were presented through MR-compatible earphones (Sensimetrics S14) at 75 dB SPL (68 dB SPL for the quieter sounds).

The visual task in Experiment 4 was a one-back task. Participants were presented a series of 4x4 grids, each with six squares filled in. Each grid was on the screen for 695 milliseconds, and there was a 122 millisecond inter-stimulus interval during which a circle in the center of the screen was shaded green if participants had given a correct response for the previous stimulus and red if not. Four stimuli were presented successively during each TR (which was 3.27 seconds in Experiment 4). The location of two of the colored squares changed from stimulus to stimulus unless the pattern repeated. Repeats occurred 30% of the time. Participants had to report these repeats with a button press during the presentation of the stimulus (i.e., within 695 milliseconds). To become familiarized with the task, participants performed a practice run of the visual task before the scan.

During all runs of Experiment 4, participants were presented both the stream of visual grids and the auditory stimuli (i.e., the visual and auditory stimuli were presented during both the visual and auditory task). The only difference between the stimuli that were presented to the subjects during the two tasks is that the participants did not receive feedback during the auditory task runs (the central visual circle simply turned green or red randomly; turning red with probability 0.1).

Acquisition

Imaging type(s)

Function MRI

Field strength

3T

Sequence & imaging parameters

MR data were collected on a 3T Siemens Trio scanner with a 32-channel head coil at the Athinoula A. Martinos Imaging Center of the McGovern Institute for Brain Research at MIT. T1-weighted anatomical images were collected in each participant (1mm isotropic voxels) for alignment and cortical surface reconstruction. In Experiments 1, 2, and 3, each functional volume consisted of twenty-one slices oriented parallel to the superior temporal plane, covering the portion of the temporal lobe superior to and including the superior temporal sulcus. Repetition time (TR) was 2.96 seconds (although acquisition time was only 560 milliseconds), echo time (TE) was 30 milliseconds, and flip angle was 90 degrees. In Experiment 4, we expanded the number of slices from twenty-one to thirty-three, so that we could be sure to acquire the entire occipital lobe in all subjects in order to record BOLD responses in visual cortical areas (i.e., to evaluate mean responses in visual cortex during the two tasks as we report in Fig. 4B). As a result, the acquisition time was increased to 870 milliseconds, and thus the TR was increased to 3.27 seconds. All other acquisition parameters were kept the same as they were in Experiment 1, 2, and 3. For each run in all four experiments, the four initial volumes were discarded to allow homogenization of the magnetic field. In-plane resolution was 2 x 2 mm (96 x 96 matrix), and slice thickness was 2.8 mm with a 10% gap, yielding an effective voxel size of 2 x 2 x 3.08 mm.

A simultaneous multislicing (SMS) factor of three was used to expedite the time of acquisition. The factor of three was selected via pilot experiments comparing protocols with different SMS factors (both higher and lower). In these pilots, we sought to maximize the degree to which responses measured from auditory cortex were reliable across presentations of the same stimulus and differentiated across presentations of different stimuli. We measured the correlation between multi-voxel response patterns to different sounds, as well as to the same sound presented multiple times. We computed the "separability" of the voxel patterns as the difference between the mean test-retest correlation (i.e., reliability) of the pattern and the mean of the correlation between patterns evoked by different pairs of stimuli. A factor of three maximized this quantity of separability. Responses measured with higher SMS factors were more similar across presentations of the same stimuli, but were also were more similar across presentations of different stimuli.

Area of acquisition

In Experiments 1, 2, and 3, each functional volume consisted of twenty-one slices oriented parallel to the superior temporal plane, covering the portion of the temporal lobe superior to and including the superior temporal sulcus. Repetition time (TR) was 2.96 seconds (although acquisition time was only 560 milliseconds), echo time (TE) was 30 milliseconds, and flip angle was 90 degrees. In Experiment 4, we expanded the number of slices from twenty-one to thirty-three, so that we could be sure to acquire the entire occipital lobe in all subjects in order to record BOLD responses in visual cortical areas (i.e., to evaluate mean responses in visual cortex during the two tasks as we report in Fig. 4B). As a result, the acquisition time was increased to 870 milliseconds, and thus the TR was increased to 3.27 seconds. All other acquisition parameters were kept the same as they were in Experiment 1, 2, and 3.

Diffusion MRI ☐ Used ☒ Not used

Preprocessing

Preprocessing software

Functional volumes were preprocessed with FreeSurfer's FSFAST and in-house MATLAB scripts. Volumes were corrected for motion and skull-stripped. Each run was aligned to the anatomical volume using FLIRT and BBRegister. These preprocessed functional volumes were then resampled to the reconstructed cortical surface. The value for each point on the surface was computed as the average of the (linearly interpolated) value at six points across the cortical ribbon: the pial boundary, the white matter boundary, and four evenly spaced locations between the two. In order to improve SNR, were smoothed on the surface with a 3mm full-width-at-half-maximum (FWHM) 2D Gaussian kernel for the ROI analyses and with a 5mm kernel for the group summary maps. All analyses were done in this surface space. The

elements in this surface space are sometimes referred to as vertices, but for ease of discussion we refer to them as voxels throughout this paper.

Normalization

For visualization purposes, subjects were aligned to FSAverage space. Registration was nonlinear and surface-based.

Normalization template

Freesurfer's FSAverage template.

Noise and artifact removal

Nuisance regressors included a linear and a quadratic regressor to account for drift and three additional regressors to help account for the residual effects of subject motion (the top three PCs of the six translation and rotation motion correction degrees of freedom).

Volume censoring

We did not censor volumes.

Statistical modeling & inference

Model type and settings

Responses to each of the 90 or 105 or 108 stimuli were estimated with a general linear model (GLM) with a unique regressor for each of the stimuli (see above for information on nuisance regressors). The hemodynamic response function was assumed to be a standard gamma function (d=2.25; t=1.25).

Effect(s) tested

To measure the robustness of each voxel's response to the presence of background noise, we computed the Pearson correlation between the voxel's response to the foreground sounds and that to the same foregrounds embedded in background noise (i.e., the mixtures). Different voxels have different levels of measurement noise (e.g., due to distance from the measurement coils), and thus to enable comparisons across voxels we corrected for this measurement noise by employing the correction for attenuation. To employ this correction, we measured the response to foregrounds and mixtures separately in even- and odd-numbered presentations (i.e., we averaged responses to the second and fourth presentations in a scanning session, and to the first and third presentations, respectively). We computed the correlation between responses to foregrounds and mixtures for the even and odd presentations, and took the average of these two correlation coefficients. We then measured the reliability of the response to the foregrounds and the reliability of the response to the mixtures, by computing the Pearson correlation between the responses to even and odd presentations of the foregrounds and mixtures, respectively. We then applied the correction for attenuation, dividing the correlation of the response to the foregrounds and mixtures by the geometric mean (square root of the product) of the reliability of the responses.

Specify type of analysis: ☐ Whole brain ☐ ROI-based ☒ Both

Anatomical location(s)

For regions of interest (ROI) analyses, we used a primary ROI defined as TE 1.1 and 1.0 and a non-primary ROI defined as the anterior and posterior superior temporal gyrus parcels from Freesurfer.

Statistic type for inference
(See Eklund et al. 2016)

We performed statistical inference on ROIs, not individual voxels.

Correction

N/A.

Models & analysis

n/a	Involved in the study
☒	☐ Functional and/or effective connectivity
☒	☐ Graph analysis
☒	☐ Multivariate modeling or predictive analysis
